# Gene Therapy Using Plasmid DNA Encoding Vascular Endothelial Growth Factor 164 and Fibroblast Growth Factor 2 Genes for the Treatment of Horse Tendinitis and Desmitis: Case Reports

**DOI:** 10.3389/fvets.2017.00168

**Published:** 2017-10-10

**Authors:** Milomir Kovac, Yaroslav A. Litvin, Ruslan O. Aliev, Elena Yu Zakirova, Catrin S. Rutland, Andrey P. Kiyasov, Albert A. Rizvanov

**Affiliations:** ^1^Moscow State Academy of Veterinary Medicine and Biotechnology, Moscow, Russia; ^2^Kazan Federal University, Kazan, Russia; ^3^Faculty of Medicine, School of Veterinary Medicine and Science, University of Nottingham, Nottingham, United Kingdom

**Keywords:** gene therapy, tendon, suspensory ligament, horse, vascular endothelial growth factor, fibroblast growth factor

## Abstract

In this clinical study, for the first time we used the direct gene therapy to restore severe injuries of the suspensory ligament branch and superficial digital flexor tendon in horses (*Equus caballus*). We injected the plasmid DNA encoding two therapeutic species-specific growth factors: vascular endothelial growth factor 164 and fibroblast growth factor 2 at the site of injury in the suspensory ligament branch and tendon. Treatment effects were evaluated with the use of clinical observation and ultrasound imaging during a period of a few months. We showed that gene therapy used within a period of 2–3 months after the injury resulted in the complete recovery of functions and full restoration of the severely damaged suspensory ligament and superficial digital flexor tendon.

## Introduction

Tendon or ligament injuries are one of the most common causes of orthopedic disorders in horses (*Equus caballus*) of any age and breed. Injuries of the digital flexor tendons (superficial and deep digital flexors) and the suspensory ligament are of utmost clinical importance in the horse resulting in more than 98% of all cases observed in practice (1). In terms of pathogenesis, a course of equine tendinitis (desmitis) can follow three phases: the inflammatory response, the *fibroblastic* phase and the *remodeling phase*, which lasts for several months and can extend up to 15 months in severe injuries (2). The main problem observed in ligament healing at the site of tendon fibril damage is the formation of granulation fibrous connective tissue (a scar) with a lot of type III collagen content—up to 30%, whereas in normal healthy tendon it is less than 5% (3). Type III collagen differs from main tendon type I collagen due to smaller sized fibers and reduced strength and elasticity.

A selection of treatment modalities are available for equine injured ligaments and tendons and their use depends upon injury type, location and extent as well as the time elapsed since the injury, type of workload that the horse undertakes, and patient age. There are conservative and surgical methods of treatment for ligament and tendon injuries in horses. Conservative treatment modalities include physiotherapy, medical therapy and regenerative medicine methods. When using medical therapy a disease relapses in more than 60% of cases upon intensive physical exercise as tendon scarring is less elastic than normal tissue (4). Therefore, recently methods of regenerative medicine have been often used to treat tendon damage. Regenerative medicine methods of treatment for tendon and ligament injuries in horses imply the use of autologous bone marrow and adipose tissue derived mesenchymal stem cells, as well as platelet-rich plasma (5, 6). These methods of treatment are successful in approximately 80–90% of cases (a return to the preinjury level of sport workload), with the relapse frequency being less than 20%, but the restoration requires more than 5–6 months (4). This is due to low blood supply of tendon and ligament tissues, decreased cell turnover, and metabolism in comparison with other tissues (for example, muscles and the skin). Therefore, healing of tendon and ligament injuries is delayed.

The use of recombinant proteins and gene therapy are the most advanced and promising approaches in the treatment of musculoskeletal disorders in human medicine (7). To date, direct gene therapy of equine tendon injuries has never been employed. In our research, we used plasmid DNA (pDNA) containing species-specific vascular endothelial growth factor (VEGF) 164 and fibroblast growth factor 2 (FGF2) genes because VEGF164 is a member of a large VEGF proteins family, which promotes proliferation and migration of endothelial cells. FGF2 is a dominant protein of FGF family. This factor shows a broad spectrum of mitogenic and angiogenic activity and is a neurotrophic factor (8). This study aimed to evaluate the therapeutic efficacy of a gene therapy pDNA containing species-specific VEGF164 and FGF2 coding sequences in the treatment of severe injury to the suspensory ligament and superficial digital flexor tendon in two horses.

## Materials and Methods

### Plasmid DNA

We have designed a pDNA-based genetic construct (named pBUDK-ecVEGF164-ecFGF2) to restore damaged connective tissue of the tendon and ligament. Recombinant plasmid contains coding sequences of *E. caballus* protein growth factors VEGF164 (also known as VEGFA164) and FGF2 (also known as bFGF). The generation of pDNA pBUDK-ecVEGF164-ecFGF2 has been previously described (8). Based on the results of agarose gel electrophoresis the resulting sterile drug preparation contained ≥95% of pDNA in a supercoiled form. Endotoxin contents were ≤0.03 EU/mg. A pDNA in lyophilized form (aliquoted in 5 mg doses) was stored at −20°C.

### Ethics

Plasmid vectors were created in accordance with the human standards set by the United States Food and Drug Administration (9, 10), and the Committee for the Medicinal Products for Human Use in the European Medicines Agency (11, 12) in order that they could be safely injected. The Institutional Review Board of the Kazan Federal University approved this study (protocol no. 3; date May 05, 2015). The horses presented at the clinic with naturally occurring injuries and informed consent given by the owners. This technique has been previously used in dogs (13) and humans (14). Injections and care were given in accordance with standard veterinary practice recommendations by qualified clinicians with additional health and welfare checks and clinical observations.

### Preparation and Administration of the pDNA

Plasmid DNA was dissolved in a tube in 5 ml of a sterile 0.9% NaCl solution to the final concentration of 1 mg/ml. The dissolved pDNA was stored at +4°C in a fridge overnight, with occasional gentle stirring and overturning. Prior to the administration into damaged tissue, the pDNA was warmed to +37°C. 3.5 ml of the pDNA solution of a total volume was filled in a syringe and under aseptic conditions and ultrasonic guidance the solution was injected into sites of injury. The filled volume was multi-injected into the site of tissue damage and adjacent normal tissues. Only a single administration of the pDNA was used to treat the tendon and ligament injury. After the pDNA administration the extremity was tightly dressed up, and the horses received stall rest for 2 days, then walking for 3 weeks as routinely prescribed for these conditions.

Ultrasonic imaging was used to evaluate the regeneration rate.

## Results

### First Case Report

**Figure 1:**
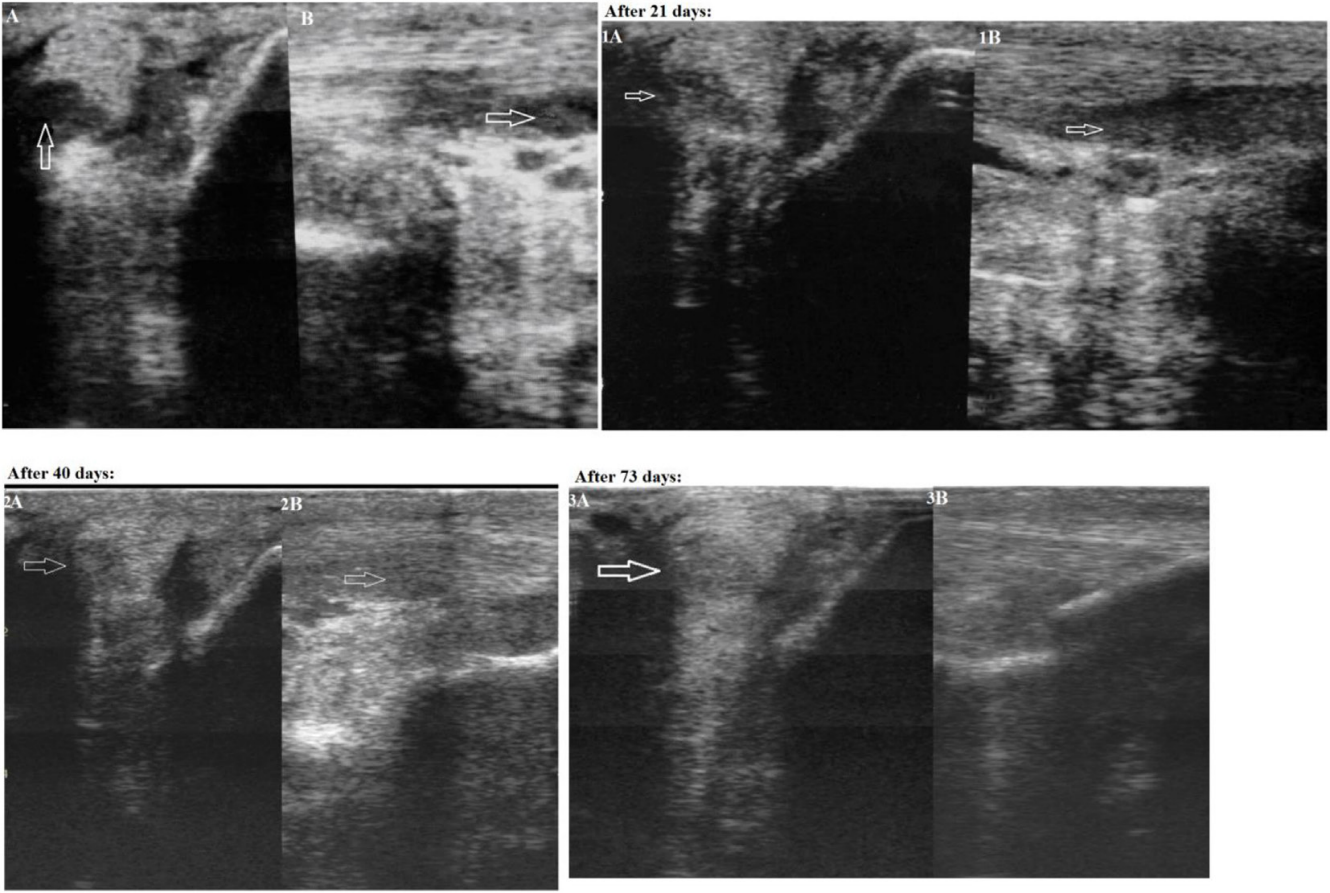
First case—transverse (Panel A) and longitudinal (Panel B) projections of the lateral crus of the suspensory ligament in zone 3C on an ultrasound image (an arrow indicates the anechoic and hypoechoic zone of the injury). Panels 1A and 1B: on Day 21 after the plasmid DNA (pDNA) was administered. Panels 2A and 2B: on Day 40 after the pDNA was administered. Panels 3A and 3B: on Day 73 after the pDNA administration.

The first case was a successful dressage 13-year-old purebred horse, male, presenting with lameness of unknown etiology for more than 10 days. Upon physical examination, it was observed that the horse was lame on the right forelimb. The lameness degree was graded as 3/5 (according to the AAEP 5-score lameness scale (2)). A marked an- and hypoechoic zone (with an area of 0.6 cm^2^) in the lateral part of the lateral crus of the suspensory ligament was visualized within area 3B-3C on an ultrasound examination of the injured limb (Figure 1: Panels A and B). The width of the lateral crus of the suspensory ligament within zone 3°C of the injured limb was 1.1 cm, and its length 1.0 cm. In addition to the anechoic and hypoechoic structure an increased cross-section volume (2.3 cm^3^) was detected on an ultrasound image of the injured ligament. A thickening of the peritendineum and accumulation of the synovial fluid within the fetlock tendon sheath were also detected. Color Doppler energy imaging revealed intensified vascularization of the injured ligament. Based on the area of injury the final diagnosis was made as “Grade 2 desmitis of the lateral crus of the suspensory ligament.”

On Day 21 after the pDNA administration, a physical examination confirmed no observable lameness when walking on firm and soft ground both in step and at trot. A flexion test of the fetlock joint was negative confirming that the horse exhibited no pain reaction. An ultrasound examination of the lateral crus performed on Day 21 after drug administration visualized an increased echogenicity in the area of injury (Figure 1: Panels 1A and 1B), indicating there were signs of moderately decreased echogenicity in the ligament part and inhomogeneity of its structure due to the formation of lysed hematoma of newly formed connective tissue —a collagen scar. Partially reduced swelling of the peritendineum could also be observed. As both clinical and ultrasound characteristics of the injured ligament had evidently improved, a gradual introduction of trotting was prescribed in addition to walking.

On Day 40 after administration of the pDNA, a physical examination showed continued signs of no lameness when walking on firm and soft ground both in step and at trot. There was a clear ultrasound improvement (increased echogenicity) of the damaged area state. A misalignment of “new” collagen fibers was visualized infrequently in this region. Tissues adjacent to the ligament (adipose layer) demonstrated normal echogenicity. A Doppler ultrasound visualized single foci of vascularization. These changes correlate with the beginning of reparative regeneration of tendon tissue, i.e., fibroplasia. As no lameness was observed, further load increasing (gallop, competitions) was prescribed. On Day 65 after pDNA injection, the horse was successful in an international dressage tournament, and displayed no signs of lameness.

On Day 73 after pDNA administration, physical examination showed that no lameness was observed when walking on firm and soft ground both in step and at trot. A further increase of echogenicity in the region of injury could be seen on an ultrasound image; however, a “scar—collagenous” change of the injured ligament was hardly visualized (Figure 1: Panels 3A and 3B). The gap between edges of the partially torn ligament was entirely filled with newly formed tissue. Two hyperechoic regions were visualized within the injured part of the ligament. A Doppler ultrasound study revealed no enhanced vascularization. The ultrasonic image conformed to almost complete recovery of the suspensory ligament.

### Second Case Report

**Figure 2:**
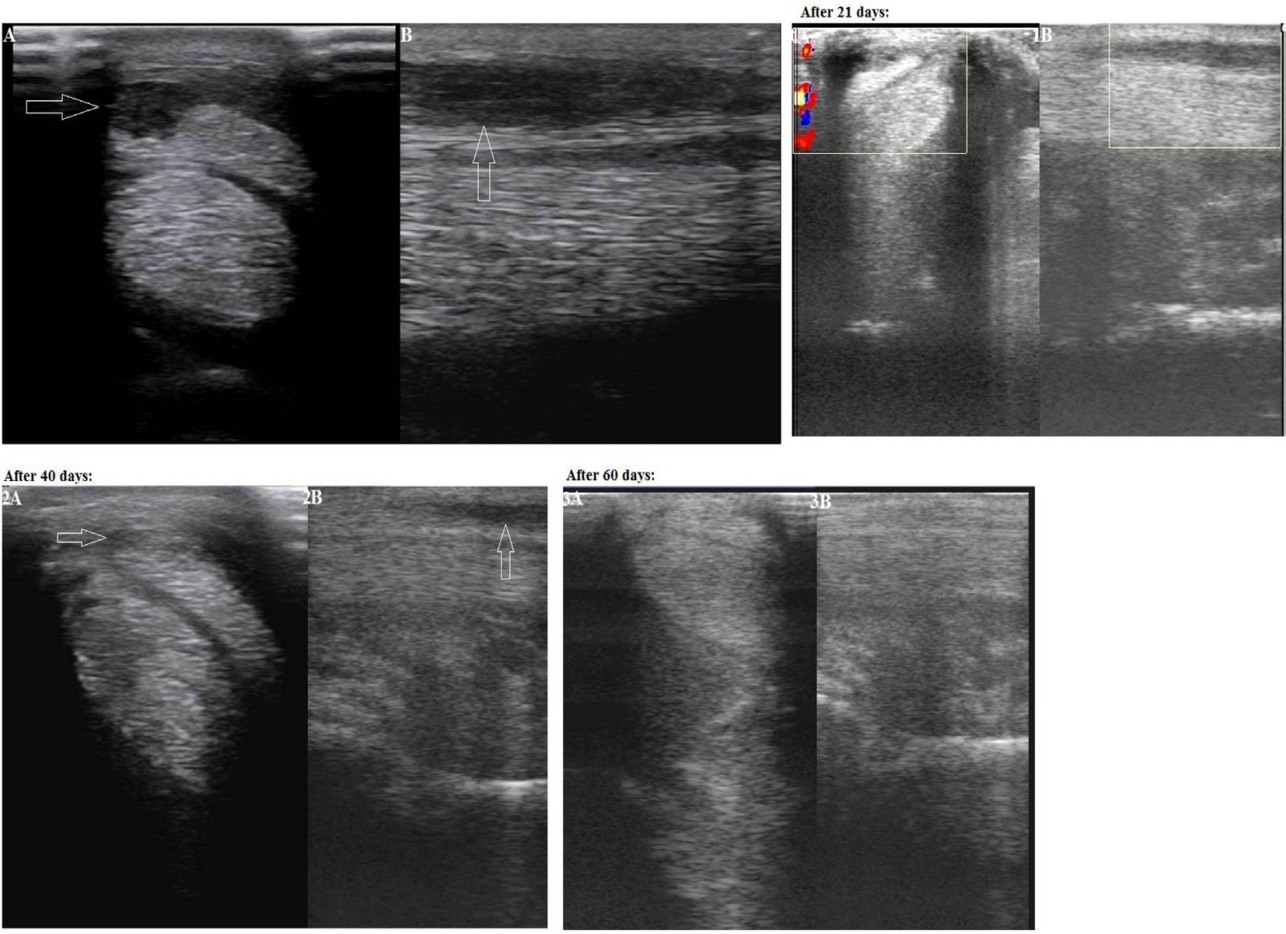
Second case—transverse (Panel A) and longitudinal (Panel B) projections of the superficial digital flexor tendon in zone 2B on an ultrasound image (an arrow indicates an anechoic and hypoechoic zone of the injury). Panels 1A and 1B: on Day 21 after the plasmid DNA (pDNA) administration. Panels 2A and 2B: on Day 40 after the pDNA administration. Panels 3A and 3B: on Day 60 after the pDNA administration.

The second case was a dressage 9-year-old half-bred Trakehner horse, male, having lameness for more than 25 days. On a physical examination, the animal lameness on the left forelimb could be seen. The lameness degree was graded as 2/5. A marked an- and hypoechoic zone (with an area of 0.9 cm^2^) of the lateral part of the superficial digital flexor tendon was visualized in zone 2B on an ultrasound examination (Figure 2: Panels A and B). The width of the superficial digital flexor tendon in this region was 0.5 cm, the length 3.8 cm, and the cross-section area 1.9 cm^2^. In addition, there was a thickening of the peritendineum. Based on the area of injury the final diagnosis in zone 2B was made as “Grade 3 tendinitis of the superficial digital flexor.”

On Day 21 following pDNA administration, physical examination showed no observable lameness when walking on firm and soft ground both in step and at trot. Increased echogenicity of the injured tendon as well as a reduction of the damage area (0.6 cm^2^) was visualized in zone 2B on an ultrasound image (Figure 2: Panels 1A and 1B). Partially reduced swelling of the peritendineum could also be observed. The clinical prescription of continued walking exercise (without trot and gallop) was given.

On Day 40 after the pDNA administration, upon physical examination no lameness was observed when walking on firm and soft ground both in step and at trot. The site of a tendon injury was hardly seen on an ultrasound image, therefore the damaged tissue had obviously improved (echogenicity increased) (Figure 2: Panels 2A and 2B). A misalignment of “new” collagen fibers was visualized in a few areas of this region. No swelling of the peritendineum was visualized at this stage in contrast to Day 21. As both clinical and ultrasound characteristics of the injured tendon had evidently improved, a gradual introduction of trotting was prescribed in addition to walking.

On Day 60 after the pDNA administration, physical examination indicated absence of lameness when walking on firm and soft ground both in step and at trot. The site of a tendon injury was hardly seen on an ultrasound image (Figure 2: Panels 3A and 3B). No misalignment of “new” collagen fibers were visualized any longer in contrast to Day 40. As no lameness was observed, further load increasing (gallop, competitions) was prescribed.

At present (more than 12 months after treatment) having received gene therapy both horses have full physical load and participate in dressage championships. Neither horse showed any adverse clinical effects to the injection.

## Discussion

Injury to the digital flexor tendon including that of the suspensory ligament is a very common orthopedic trauma in sporting horses which in a significant number of cases results in not only temporary lameness but in a relapse of the disease with an early termination of a competitive career of such animals.

Gene therapy, as one of the most advanced technologies in medicine, besides promising therapy of hereditary diseases, offers novel opportunities for the clinical treatment of numerous orthopedic disorders including injuries of the tendon and ligament (7, 15, 16). In this study, horse tendinitis and desmitis treatment using injected pDNA encoding species-specific VEGF164 and FGF2 cDNAs resulted in a return of animals to their preinjury level of sports load, compared with traditional medical and regenerative therapies (4). We used VEGF164 and FGF2 genes because proteins encoding these genes influence on the process of regeneration of the vessels and connective tissue at organism equine. VEGF164 *in vivo* attracts endothelial progenitor cells from the bone marrow and stimulates angiogenesis. Further, it enhances the activity of pericytes and stabilizes newly formed vessels, increases vascular permeability at the site of injury, which promotes the formation of granulation tissues. FGF2 stimulates proliferation of cells, regeneration of nervous, muscular, and connective tissue.

It should be emphasized that the lameness disappearance after treatment of tendinitis in a horse does not mean absolute tissue regeneration. Intensive training of a horse can be integrated into the training once ultrasound examinations confirm a “complete” healing of the injured tendon or ligament. In our cases, a rapid complete regeneration of both the tendon and the ligament occurred within 2–3 months of treatment and was observed after treatment with pBUDK-ecVEGF164-ecFGF2 pDNA by measuring echogenicity and an area of injury, the percentage of parallel collagen fibers.

The exact mechanisms of action behind the rapid effects of direct gene therapy with pBUDK-ecVEGF164-ecFGF2 on healing the injured equine ligament and tendon are yet to be determined as the horses made a full recovery and no further histological interventions would have been appropriate. Based on some previous studies, growth factors VEGF and FGF2 are polypeptides which are primarily released by platelets, fibroblasts, and endothelial cells at the site of injury (17). Their mechanisms of action are complex and closely related to other factors of inflammation and regeneration. They are considered to stimulate migration of tendoblasts, fibroblasts, and mesenchymal stem cells which are responsible for production of collagen and other constituents of extracellular components of ligaments and tendons such as proteoglycans, glycosaminoglycans, and glycoproteins, *via* enhanced angiogenesis (the process of new blood vessel formation) at a site of injury (7).

The use of direct gene therapy with these growth factors is highly promising for the treatment of orthopedic disorders not only in horses but in other animal species and humans. The application of direct gene therapy with a similar plasmid construct based on dog-specific VEGF164 and bone morphogenetic protein 2 genes for the treatment of an injury of the anterior cruciate ligament in a large breed dog has been reported previously (13). Moreover, in human clinical practice gene therapy pDNA encoding VEGF and FGF2 genes were used for the treatment of patients with critical lower limb ischemia (14). Finally, pDNA pl-VEGF165, encoding human VEGF165, demonstrated safety and efficacy in patients with chronic lower limb ischemia and was approved in Russia for the treatment of atherosclerotic peripheral arterial disease (18, 19). The high efficacy and safety of the direct pDNA gene therapy has been demonstrated in all of these cases.

The most recent studies both *in vitro* and *in vivo* have demonstrated that direct FGF2 (bFGF) and VEGF-based gene therapy with an adenoassociated viral vector 2 in chicken flexor tendons resulted in significantly improved tendon strength and elasticity by increasing production of type I collagen and other extracellular tendon molecules evidently as well as by accelerating cell proliferation (16).

Reported method of using direct gene therapy for the treatment of tendon and ligament injuries in horses is novel, however, it requires further research to proof safety and efficiency. A complete effect of direct VEGF and FGF2 gene therapy on the healing of injured tendons and ligaments in horses cannot be evaluated without involving a large number of experimental animals and methods of a double-blind study. To obtain a more comprehensive picture a histological examination and immunohistochemistry of biopsy materials obtained from an injured tendon are required. These methods include a thorough analysis of the tissue-specific expression of a relevant gene, identification, density, and concentration of type I collagen, an assay of the structure, function, and intracellular distribution of its protein product as well as biochemistry of the pathological process. There are still a great number of challenges are to be solved before gene therapy will be widely used in clinical practice of veterinary medicine, but this study makes advances in the use of these therapies. Since these preliminary case reports demonstrated improvement in clinical outcome and no adverse side effects, larger clinical trial is ongoing to further study efficiency of direct gene therapy for the treatment of horse tendinitis and desmitis.

## Author Contributions

MK: medical diagnosis, clinical observation of horses, pDNA administration, design of the study (clinical part), and writing the manuscript. YL: purification clinical grade pDNA for gene therapy, assistance in pDNA administration, design of the study, collection of data and interpretation of results, and writing the manuscript. RA: collection and interpretation of clinical data. EZ: testing of pDNA efficiency and editing the manuscript. CR and AK: intellectual contribution into the discussion and writing the manuscript. AR: design of the study, interpretation of results and intellectual contribution into the discussion and writing the manuscript.

## Conflict of Interest Statement

The authors declare that the research was conducted in the absence of any commercial or financial relationships that could be construed as a potential conflict of interest.
